# 干扰FOXC1逆转非小细胞肺癌吉非替尼耐药的作用

**DOI:** 10.3779/j.issn.1009-3419.2021.103.11

**Published:** 2021-08-20

**Authors:** 聪 彭, 盼 李, 明强 杨, 丹扬 陈, 渊锋 黄

**Affiliations:** 1 510095 广州，广州医科大学附属肿瘤医院病理科 Department of Pathology, Affiliated Cancer Hospital of Guangzhou Medical University, Guangzhou 510095, China; 2 510095 广州，广州医科大学附属肿瘤医院肿瘤研究所 Caner Research Institute, Affiliated Cancer Hospital of Guangzhou Medical University, Guangzhou 510095, China; 3 510095 广州，广州医科大学附属肿瘤医院胸外科 Department of Thoracic Surgery, Affiliated Cancer Hospital of Guangzhou Medical University, Guangzhou 510095, China

**Keywords:** 肺肿瘤, FOXC1, 吉非替尼耐药, 肿瘤干细胞, Lung neoplasms, FOXC1, Gefitinib resistance, Cancer stem cells

## Abstract

**背景与目的:**

肺癌是我国发病率和死亡率最高的恶性肿瘤，其中非小细胞肺癌(non-small cell lung cancer, NSCLC)约占肺癌的80%。表皮生长因子受体酪氨酸激酶抑制剂(epidermal growth factor receptor-tyrosine kinase inhibitor, EGFR-TKI)靶向治疗已成为NSCLC临床治疗的主要手段，然而不可避免的耐药性出现极大限制了EGFR-TKI的治疗效果。叉头框蛋白C1(forkhead box protein C1, FOXC1)是叉头框蛋白家族重要成员，在NSCLC异常表达并参与调控NSCLC进展。本研究旨在探讨干扰FOXC1对NSCLC吉非替尼耐药的影响及可能机制。

**方法:**

应用Western blot、免疫组化方法检测FOXC1在NSCLC吉非替尼耐药细胞和组织中的表达情况; 应用FOXC1 shRNA转染HCC827/GR细胞，筛选稳定干扰FOXC1的HCC827/GR细胞，采用新型四氮唑盐比色法(Methyl-thiazolyldiphenyl-sulfophenyl-tetrazolium bromide assay, MTS)法、流式细胞术及微球体形成实验检测细胞增殖、细胞凋亡及细胞自我更新能力; 采用实时荧光定量PCR(quantitative real-time PCR, qRT-PCR)、Western blot检测干性标志物SOX2、Nanog、OCT4和CD133的表达水平; 运用流式细胞术检测CD133的表达情况; 免疫组化检测耐药组织中SOX2和CD133的表达情况; 基于癌症基因组图谱(The Cancer Genome Atlas, TCGA)数据库的肺腺癌数据集，分析FOXC1、SOX2和CD133表达的相关性。

**结果:**

FOXC1在NSCLC吉非替尼耐药细胞HCC827/GR的表达水平显著高于HCC827敏感细胞(*P* < 0.05)，免疫组化结果显示FOXC1在NSCLC吉非替尼耐药组织中高表达。与对照组相比，稳定干扰FOXC1的HCC827/GR细胞对吉非替尼的半数抑制浓度(50% inhibitory concentration, IC_50_)值显著降低(*P* < 0.01)，细胞增殖能力降低、凋亡率升高(*P* < 0.05)。干扰FOXC1能够抑制SOX2、CD133的表达，并减弱HCC827/GR耐药细胞的微球体形成能力。免疫组化结果显示，吉非替尼耐药组织中SOX2和CD133表达显著高于敏感组织(*P* < 0.01)。相关性分析结果显示，FOXC1、SOX2和CD133表达两两呈正相关(*P* < 0.05)。

**结论:**

FOXC1参与NSCLC吉非替尼耐药，其机制可能与FOXC1调控肿瘤干细胞(cancer stem cells, CSCs)特性有关。

## Abstract (en)

**Background and objective:**

Lung cancer is the malignant tumor with the highest incidence and mortality in China, among which non-small cell lung cancer (NSCLC) accounts for about 80%. Epidermal growth factor receptor-tyrosine kinase inhibitor (EGFR-TKI) targeted therapy has been playing an important role in treatment of NSCLC. However, unavoidable therapeutic resistance significantly limits the clinical efficacy of EGFR-TKI. As a key member of the forkhead box protein family, FOXC1 is aberrantly expressed in NSCLC and involved in NSCLC progression. The aim of this work is to investigate the effect and potential mechanism of FOXC1 on gefitinib resistance in NSCLC.

**Methods:**

Western blot was performed to assess the expression of FOXC1 protein in HCC827/GR cells. Immunohistochemistry (IHC) assays were performed in human NSCLC tissues with gefitinib resistance. HCC827/GR cells were transfected with shRNA specifically targeting FOXC1 mRNA and stable cell lines were established. The effects of FOXC1 on cell viability and apoptosis were analyzed using a new methyl thiazolyl tetrazolium assay (MTS assay) and flow cytometry. Self-renewal ability was determined by mammosphere-formation analysis. Quantitative real-time PCR (qRT-PCR) and Western blot were employed to detect the expression of SOX2, Nanog, OCT4 and CD133. Flow cytometry analysis were further used to detect the level of CD133. IHC assays were used to detect the levels of SOX2 and CD133 in NSCLC tissues with genfitiinb resistance. Correlations of the expressions of FOXC1, CD133 and SOX2 with each other in lung adenocarcinoma samples were analyzed based on The Cancer Genome Atlas (TCGA) database.

**Results:**

The expression of FOXC1 is significantly increased in HCC827/GR cells compared with HCC827 cells (*P* < 0.05). IHC results showed FOXC1 was highly expressed in NSCLC tissues with gefitinib resisitance. Knockdown of FOXC1 significantly increased the sensitivity of HCC827/GR cells to gefitinib. The cell viability was decreased and the apoptosis was promoted (*P* < 0.05). Moreover, FOXC1 knockdown apparently inhibited the expression of SOX2 and CD133, and decreased the mammosphere-formation capacity in HCC827/GR cells. In NSCLC tissues with gefitinib resistance, the expressions of SOX2 and CD133 were significantly higher compared with gefitinib-sensitive tissues (*P* < 0.01). Meanwhile, the expressions of FOXC1, CD133 and SOX2 with each other were positively correlated (*P* < 0.05).

**Conclusion:**

FOXC1 could increase gefitinib resitance in NSCLC, by which mechanism is related to the regulation of cancer stem cell properties.

肺癌的发病率和死亡率均居我国恶性肿瘤首位^[1]^。肺癌依据其组织形态和临床特征可分为小细胞肺癌(small cell lung cancer, SCLC)和非小细胞肺癌(non-small cell lung cancer, NSCLC)，NSCLC占肺癌的80%左右^[2]^。表皮生长因子受体(epidermal growth factot receptor, *EGFR*)属于跨膜受体酪氨酸激酶家族，是NSCLC最重要的驱动基因，靶向EGFR已经成为NSCLC临床治疗的主要手段之一^[3]^。以吉非替尼(Gefitinib)为代表的EGFR小分子抑制剂已成功应用于NSCLC患者的临床治疗，可显著延长患者无进展生存期^[4]^。然而，大部分接受治疗的患者在治疗9个月-12个月后不可避免会出现程度不同的治疗耐受，使得NSCLC总生存率难以获得明显提高^[5]^。因此，探索EGFR-TKIs耐药机制、寻找逆转耐药的途径成为了亟待解决的问题。

叉头框蛋白C1(forkhead box protein C1, FOXC1)是叉头框蛋白家族重要成员，参与正常的胚胎发育^[6]^。研究^[7, 8]^表明FOXC1在多种肿瘤中异常表达，在肿瘤发展和转移中起关键作用。研究发现FOXC1在NSCLC高表达，且与患者的预后不良密切相关^[9]^，敲除FOXC1能够抑制NSCLC的增殖、侵袭和转移^[10]^。然而，迄今对于FOXC1在NSCLC EGFR-TKI靶向治疗耐药中的作用及机制尚不明确。本研究拟探讨FOXC1促进NSCLC吉非替尼耐药的作用及可能机制。

## 1 材料与方法

### 1.1 材料

RPMI-1640培养基、胎牛血清、胰蛋白酶、Trizol试剂(Thermo Fisher公司); FOXC1、SOX2、Nanog、β-actin抗体以及HRP标记山羊抗鼠IgG、山羊抗兔IgG(CST公司); Annexin-APC细胞凋亡检测试剂盒(联科生物公司); RIPA裂解液(碧云天生物技术公司); ECL化学发光底物试剂盒(Pierce公司); Quick Start Bradford蛋白定量试剂(Bio-Rad公司); 反转录试剂盒、实时荧光定量PCR(PrimeScript RT-PCR)试剂盒(TaKaRa公司); PCR引物(上海生工公司); Gefitinib、嘌呤霉素(Sigma公司)，其余试剂均为国产分析纯以上。

### 1.2 细胞培养

人NSCLC吉非替尼敏感细胞株HCC827及其吉非替尼耐药细胞株HCC827/GR均由广州医科大学附属肿瘤医院研究所保藏。采用RPMI-1640培养基(含10%胎牛血清、100 U/mL青霉素、100 U/mL链霉素)，于37 ℃、5%CO_2_的培养箱内饱和湿度培养。

### 1.3 FOXC1稳定干扰细胞系和过表达细胞系的筛选

FOXC1 shRNA干扰质粒购自吉凯基因，FOXC1 shRNA干扰质粒及对照质粒的靶向序列分别为：shFOXC2-1：5'-CCAGTGCAGCATGCGAGCGAT-3';shFOXC2-2：5'-CCAGTGCAGCATGCGAGCGAT-3';shControl：5'-AGAACATCATGACCCTGCGAA-3'。pCMV-FOXC1过表达质粒购自Origene公司。取对数生长期的细胞，接种于6孔细胞培养板中，37℃、5%CO_2_的培养箱中培养过夜。根据Lipofectamine 2000转染试剂说明书，将相应的质粒转染至细胞中。培养48 h后，采用嘌呤霉素分别筛选稳定干扰FOXC1和稳定过表达FOXC1的细胞株。

### 1.4 新型四氮唑盐比色法检测(Methyl-thiazolyldiphenyl-sulfophenyl-tetrazolium bromide assay, MTS)实验

取对数生长期的HCC827及HCC827/GR细胞，胰蛋白酶消化后制成单细胞悬液，以1×10^4^个/mL接种细胞到96孔板中培养过夜。向对应试验孔中加入不同浓度的吉非替尼，培养72 h后，每孔加入20 μL MTS溶液(Promega公司)，继续培养4 h。采用酶标仪测定490 nm波长下的光密度(optical density, OD)值。每组浓度设立3个复孔，独立重复实验3次。

### 1.5 细胞凋亡检测

以3×10^5^个/孔接种HCC827及HCC827/GR细胞至6孔板，培养过夜，加入吉非替尼处理细胞48 h。收集细胞，按照Annexin V-APC/7-AAD凋亡检测试剂盒(联科生物公司)的说明书，先后加入Annexin V-APC和7-AAD，避光、室温孵育10 min后，流式细胞仪检测细胞凋亡情况。

### 1.6 实时荧光定量PCR

利用Trizol试剂提取细胞总RNA，按Primescript RT reagent Kit反转录试剂盒说明书将RNA逆转录为cDNA，Real-time PCR反应体系及条件参照SYBR Premix Ex TapTM试剂盒(TaKaRa)，2^-△△CT^法计算mRNA的相对表达量，以GAPDH作为内参。每个实验组重复3次。SOX2上游引物序列：5'-CCCACCTACAGCATGTCCTACTC-3'，下游引物序列：5'-TGGAGTGGGAGGAAGAGGTAA-3';Nanog上游引物序列：5'-TTCCCTCCTCCATGGATCTG-3'，下游引物序列：5'-TGTTTCTTGACTGGGACCTTGTC-3';OCT4上游引物序列：5'-TTCAGCCAAACGACCATCTG-3'，下游引物序列：5'-CACGAGGGTTTCTGCTTTGC-3';CD133上游引物序列：5'-TTACGGC-ACTCTTCACCT-3'，下游引物序列：5'-TATTCCACAA-GCAGCAAA-3';GAPDH上游引物序列：5'-GCACCGTCAAGGCTGAGAAC-3'，下游引物序列：5'-TGGTGAAGACGCCAGTGGA-3'。

### 1.7 Western blot

PBS洗涤细胞2次，加入RIPA裂解液冰上放置30 min，收集裂解液，4 ℃ 12, 000 rpm离心20 min，取上清，采用BCA法测定蛋白质浓度。等量蛋白质样品经10%的SDS-PAGE电泳分离后，转印至PVDF膜，5%脱脂奶粉室温封闭1 h，加入一抗FOXC1(1:1, 000稀释)，SOX2(1:1, 000稀释)，Nanog(1:1, 000稀释)和β-actin(1:1, 000稀释)，4℃孵育过夜; PBST洗涤3次，每次10 min，加入HRP标记的特异性二抗(1:5, 000稀释)，室温孵育1 h; PBST洗涤3次，每次10 min，ECL化学发光检测显影，凝胶成像系统拍照。

### 1.8 微球体形成实验

配制微球体培养基：DMEM/F12培养基(含20 ng/mL EGF、10 ng/mL bFGF及10 μL/mL B27)，取对数生长期的细胞，胰蛋白酶消化后制成单细胞悬液，以1×10^3^/mL细胞接种至低粘附的6孔板，每孔2, 000个细胞，放37℃、5%CO_2_的培养箱中培养，每4天加适量的培养基，2周后观察拍照，计算微球体形成的个数。

### 1.9 免疫组化实验

选取2016年1月-2018年12月我院收治的*EGFR*基因突变NSCLC并采用EGFR-TKIs药物作为一线治疗的耐药患者15例，并选取同期于我院治疗的非耐药患者15例为对照组。

病理组织切片经脱蜡和水化处理，采用0.3%过氧化氢阻断内源过氧化物酶活性，用0.1 mmol/L柠檬酸盐缓冲液进行高压修复。山羊血清室温下封闭1 h，加入FOXC1抗体4 ℃孵育过夜; PBS洗涤3次，二抗室温孵育30 min，PBS洗涤3次; DAB显色、苏木素复染之后，脱水封片。按半定量积分方法，每例均随机观察5个高倍视野(×400)，相差显微镜下评估FOXC1的表达。按阳性细胞比例计分：≤25%为1分，26%-50%为1分，51%-75%为2分，≥76%为3分; 按照染色强度分别计0分-3分; 两者乘积为FOXC1免疫组化得分：0分-2分为阴性，3分-5分为阳性，6分-9分为强阳性。

### 1.10 统计学分析

所有实验均重复3次，数据以平均数±标准差(Mean±SD)表示，采用GraphPad Prism 7.0软件进行分析，组间差异比较采用*t*检验分析。采用*Pearson* *r*检验分析基因表达的相关性。*P* < 0.05具有统计学差异。

## 2 结果

### 2.1 FOXC1在HCC827/GR细胞和耐药组织中高表达

首先，采用MTS实验对人NSCLC细胞的耐药性进行分析，结果如图 1A所示，相比HCC827敏感细胞，吉非替尼对耐药细胞HCC827/GR的增殖抑制作用明显减弱(*P* < 0.05)。采用Western blot检测FOXC1在吉非替尼耐药细胞HCC827/GR中的表达情况，结果发现FOXC1在HCC827/GR细胞中的表达水平显著高于其在HCC827细胞中的表达水平(*P* < 0.05，图 1B)。免疫组化检测FOXC1在NSCLC吉非替尼耐药组织和敏感组织中的表达情况，结果发现FOXC1在吉非替尼耐药组织中的表达高于敏感组织(图 1C，图 1D)，差异有统计学意义(*P*=0.042, 3)。

**图 1:**
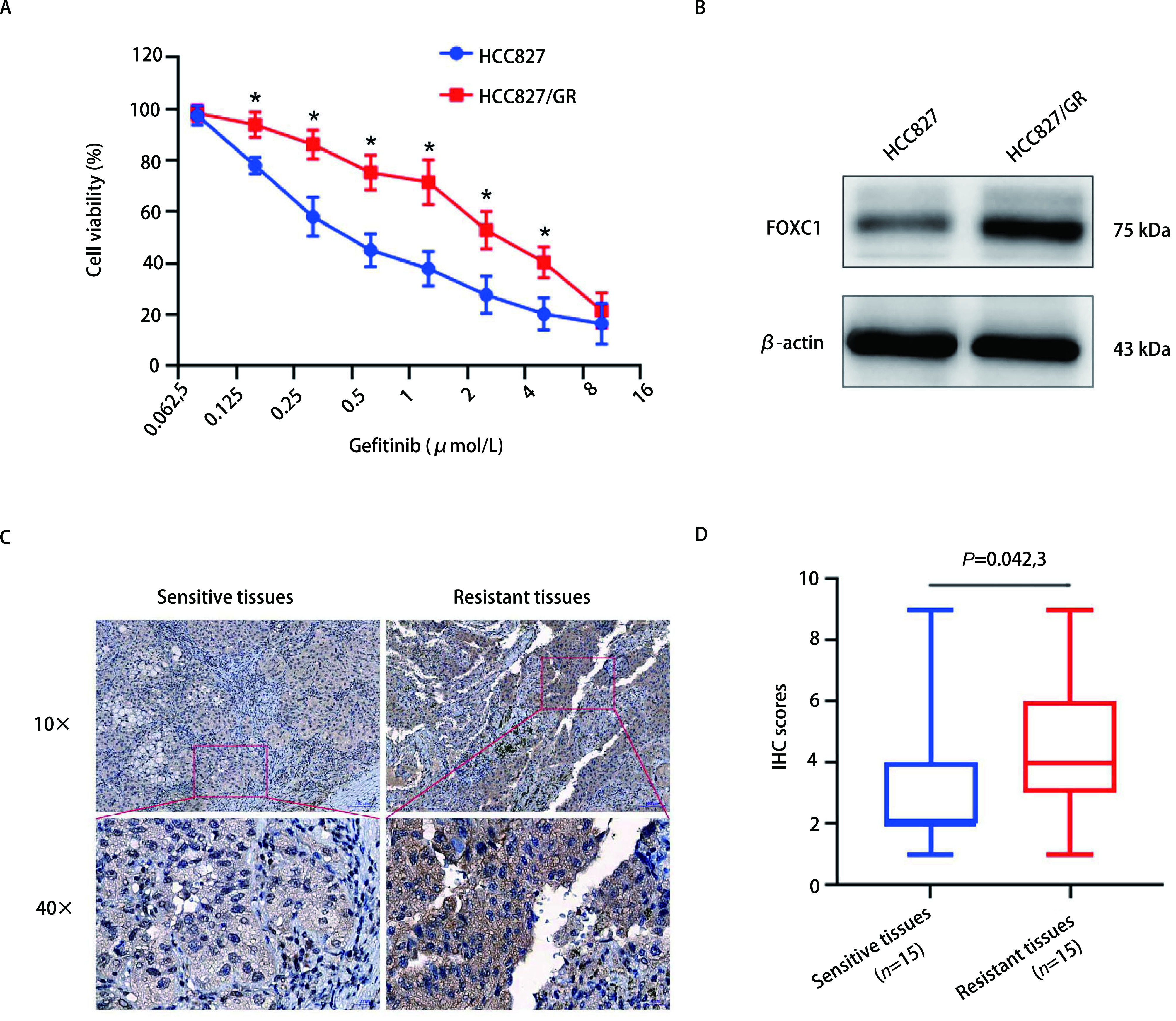
FOXC1在吉非替尼耐药细胞和组织中高表达。A: MTS实验检测HCC827/GR细胞的增殖能力(^*^P < 0.05);B: Western blot检测FOXC1在HCC827/GR细胞的表达情况; C: 免疫组化检测FOXC1在NSCLC吉非替尼耐药组织中的表达情况; D: 箱线图分析FOXC1在NSCLC耐药组织表达的得分情况。 FOXC1 is over-expressed in HCC827/GR cells and human NSCLC tissues with gefitinib-resistance. A: MTS assays were used to detect cell viability of HCC827/GR cells (^*^P < 0.05); B: Western blot was used to detect FOXC1 expression in HCC827/GR cells; C: IHC assays were performed in human NSCLC tissues with gefitinib resistance; D: Box-plot showed that FOXC1 expression scores in human NSCLC tissues with gefitinib resistance.

### 2.2 干扰FOXC1增加HCC827/GR细胞对吉非替尼的敏感性

为考察FOXC1是否介导NSCLC吉非替尼耐药，采用两个FOXC1 shRNA干扰质粒分别干扰HCC827/GR耐药细胞中FOXC1的表达。Western blot结果显示两个干扰质粒均能显著下调HCC827/GR耐药细胞中FOXC1的表达(图 2A)。进一步采用MTS分析干扰FOXC1对吉非替尼耐药性的影响，结果发现，干扰FOXC1的HCC827/GR细胞在吉非替尼的诱导下的增殖能力较HCC827/GR细胞有所降低(*P* < 0.05，图 2B)。干扰FOXC1后的HCC827/GR细胞的半数抑制浓度(50% inhibitory concentration, IC_50_)值较对照组细胞显著降低(*P* < 0.01，图 2C)。同时，MTS检测分析稳定过表达FOXC1的HCC827细胞。结果如图 2E所示，与对照组细胞相比，过表达FOXC1的HCC827细胞在吉非替尼诱导下的增殖能力有所提高(*P* < 0.05);过表达FOXC1的HCC827细胞IC_50_值较对照组细胞显著升高(*P* < 0.01，图 2F)。上述结果表明，干扰FOXC1能明显增加HCC827/GR耐药细胞对吉非替尼的敏感性。

**图 2:**
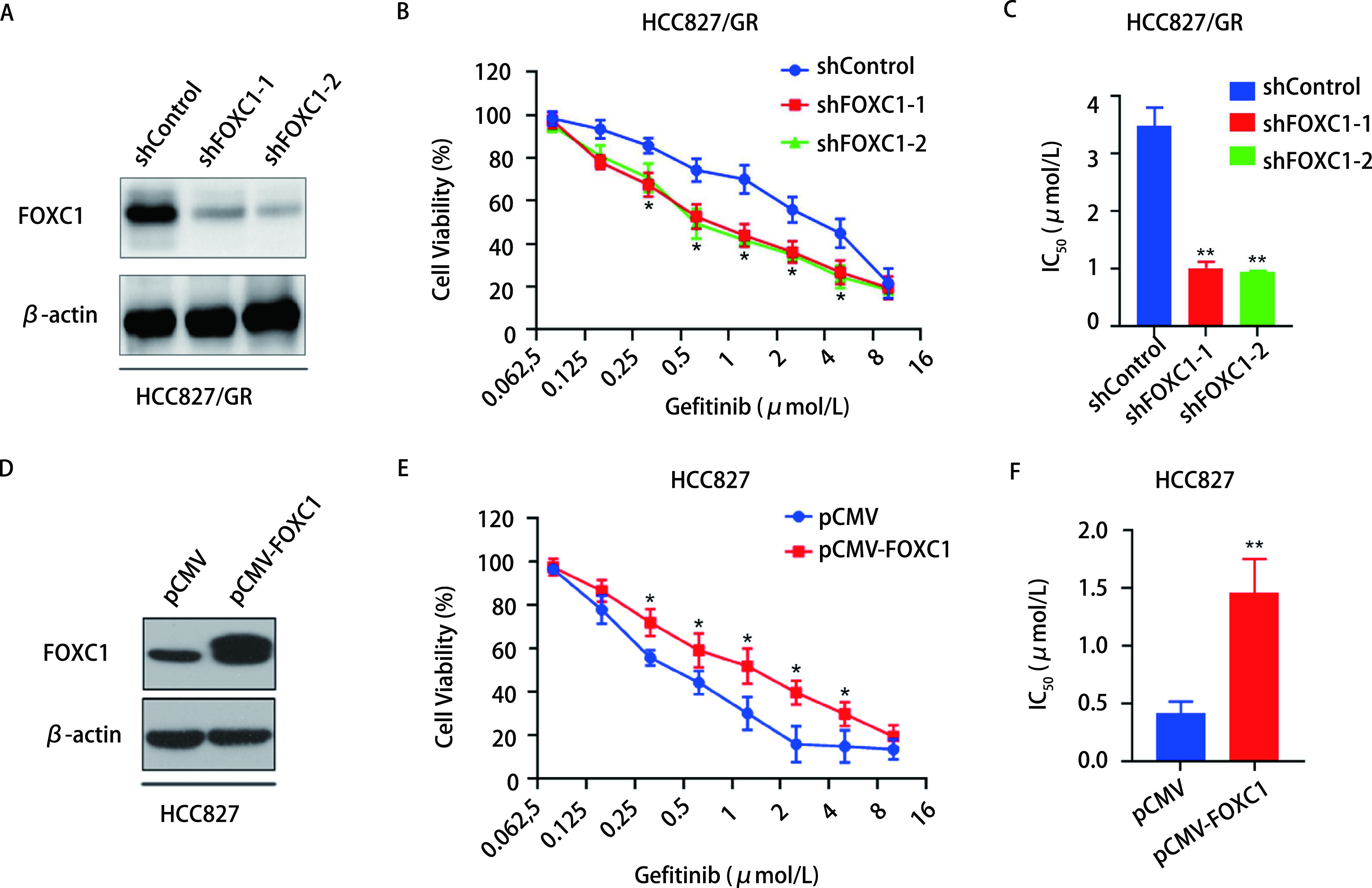
干扰FOXC1增加HCC827/GR细胞对吉非替尼的敏感性。A: Western blot验证FOXC1的干扰效果; B: MTS实验检测干扰FOXC1对HCC827/GR细胞增殖能力的影响(^*^P < 0.05);C: HCC827/GR细胞IC_50_值的计算(^**^P < 0.01);D: Western blot验证FOXC1的过表达效果; E: MTS实验检测过表达FOXC1对HCC827细胞增殖能力的影响(^*^P < 0.05);F: HCC827细胞IC_50_值的计算(^**^P < 0.01)。 Knockdown of FOXC1 increased gefitinib sensitivity of HCC827/GR cells. A: Western blot was used to detect the interference effect of FOXC1 shRNA; B: MTS assays were used to detect cell viability of HCC827/GR cells with FOXC1 knockdown (^*^P < 0.05); C: The IC_50_ of HCC827/GR cells were calculated (^**^P < 0.01); D: Western blot was used to detect the expression of FOXC1; E: MTS assays were used to detect cell viability of FOXC1-overexpressing HCC827 cells (^*^P < 0.05); F: The IC_50_ of HCC827 cells were calculated (^**^P < 0.01). MTS: Methyl-thiazolyldiphenyl-sulfophenyl-tetrazolium bromide; IC_50_: 50% inhibitory concentration.

### 2.3 干扰FOXC1增加吉非替尼诱导的HCC827/GR细胞凋亡

为明确FOXC1对吉非替尼耐药细胞凋亡的作用，采用Annexin V-APC/7-AAD凋亡试剂盒检测干扰FOXC1的HCC827/GR细胞凋亡情况。如图 3A和图 3B所示，在无吉非替尼诱导的情况下，干扰FOXC1没有对HCC827/GR细胞凋亡产生影响; 而HCC827/GR细胞经浓度为3 μmol/L吉非替尼的处理，干扰FOXC1使耐药细胞的凋亡比率明显升高(*P* < 0.05)。上述结果表明，干扰FOXC1能够增加吉非替尼诱导的HCC827/GR细胞凋亡。

**图 3:**
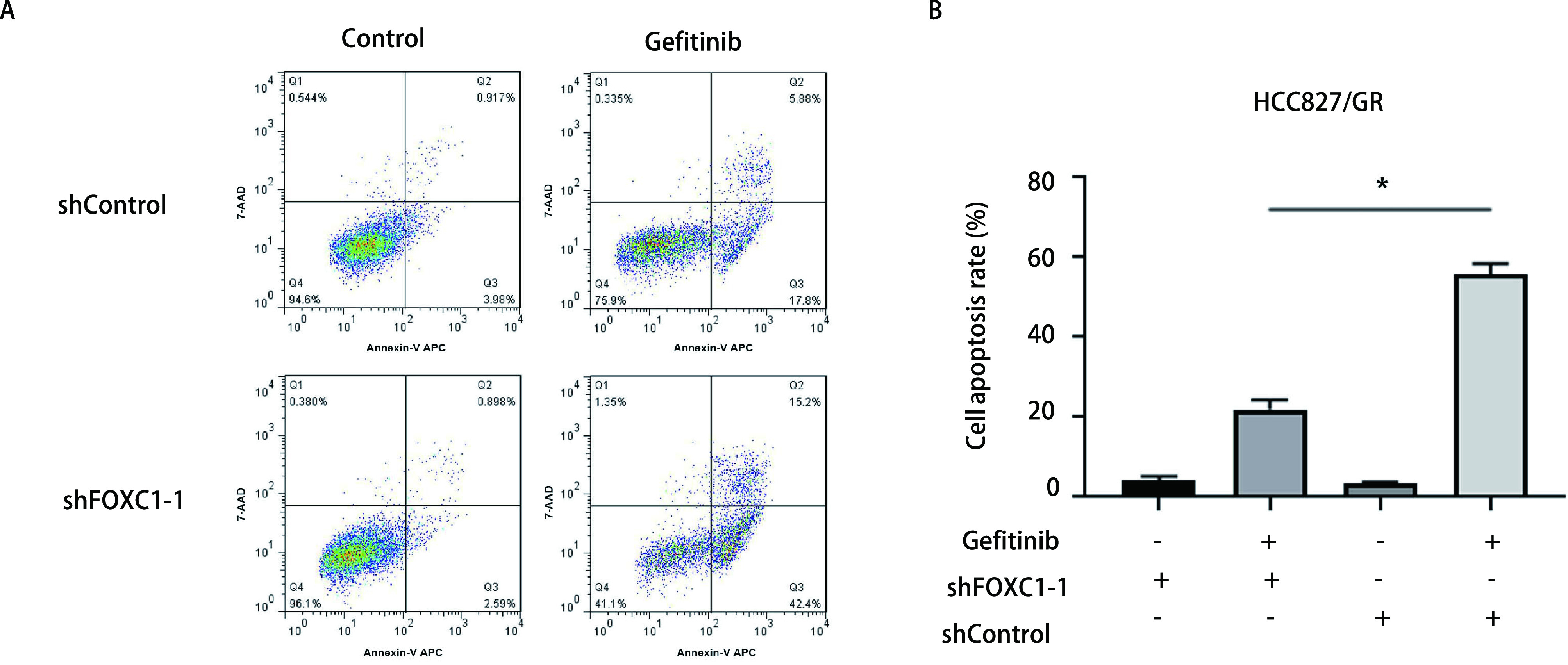
干扰FOXC1增加吉非替尼诱导的HCC827/GR细胞凋亡。A: 流式细胞术检测干扰FOXC1对HCC827/GR细胞凋亡的影响; B: 柱状图分析干扰FOXC1后HCC827/GR细胞发生凋亡的比例(^*^P < 0.05)。 Knockdown of FOXC1 increased gefitinib-induced cell apoptosis of HCC827/GR cells. A: The effect of FOXC1 knockdown in HCC827/GR cells on apoptosis was analyzed by flow cytometry; B: The histogram analysis showed the proportion of apoptosis cells (^*^P < 0.05).

### 2.4 干扰FOXC1抑制HCC827/GR细胞的肿瘤干细胞特性

已有研究表明，获得肿瘤干细胞(cancer stem cells, CSCs)特性是EGFR-TKI获得性耐药的重要机制^[11]^。qRT-PCR结果显示HCC827/GR细胞中CSCs标志物CD133的mRNA表达量明显高于敏感细胞(*P* < 0.05，图 4A)，而干扰FOXC1能够抑制HCC827/GR细胞CD133 mRNA的表达(图 4B)。流式细胞术检测结果分析可见，干扰FOXC1能下调CD133的蛋白表达水平(图 4C)。紧接着，采用微球体形成实验分析FOXC1对HCC827/GR细胞微球体形成的影响，从而判断其干细胞特性之自我更新能力的变化。结果发现，干扰FOXC1的HCC827/GR细胞中微球体形成数量少于对照组(*P* < 0.05，图 4D，图 4E)。上述结果提示，干扰FOXC1抑制HCC827/GR细胞的干细胞特性。

**图 4:**
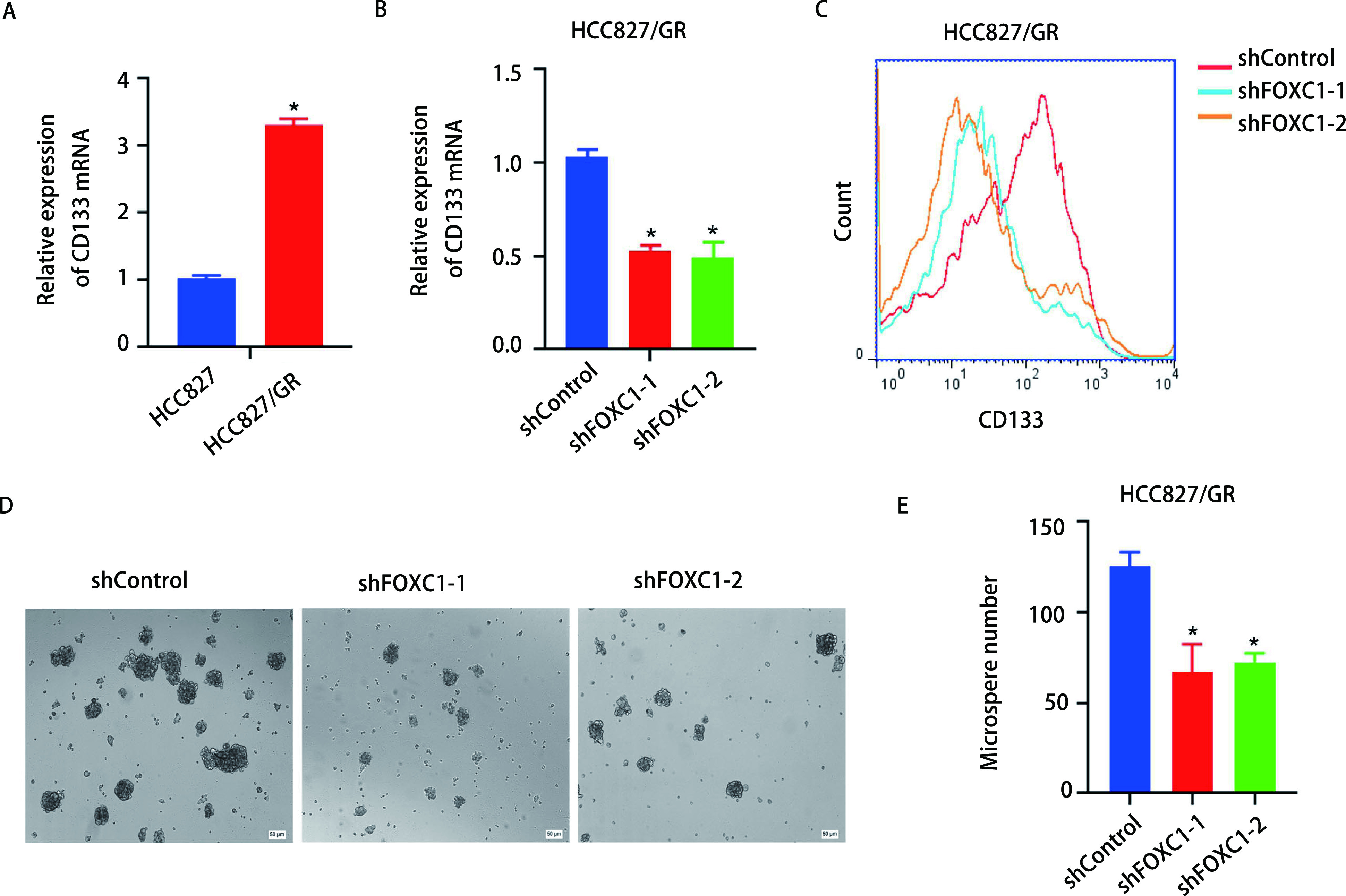
干扰FOXC1抑制HCC827/GR细胞的肿瘤干细胞特性。A：qRT-PCR检测HCC827/GR细胞CD133的mRNA表达水平(^*^P < 0.05);B：qRT-PCR检测干扰FOXC1后HCC827/GR细胞CD133的mRNA表达水平(^*^P < 0.05);C：流式细胞术检测干扰FOXC1后HCC827/GR细胞CD133的蛋白表达情况; D：微球体形成实验分析干扰FOXC1对HCC827/GR细胞干细胞特性之自我更新能力的影响; E：柱状图分析微球体形成数量(^*^P < 0.05)。 Knockdown of FOXC1 inhibited cancer stem cell properties of HCC827/GR cells. A: The mRNA level of CD133 in HCC827/GR cells was detected by qRT-PCR (^*^P < 0.05); B: The mRNA level of CD133 in HCC827/GR cells with FOXC1 knockdown was detected by qRT-PCR (^*^P < 0.05); C: The protein level of CD133 in HCC827/GR cells with FOXC1 knockdown was analyzed by flow cytometry; D: The stem cell self-renewal of HCC827/GR cells with FOXC1 knockdown was detected by microsphere formation experiments; E: The histogram analysis showed the number of microspheres (^*^P < 0.05). qRT-PCR: quantitative real-time PCR.

### 2.5 干扰FOXC1抑制HCC827/GR细胞SOX2的表达

为明确FOXC1如何抑制HCC827/GR细胞的干细胞特性，我们首先检测了CSCs的转录因子SOX2、OCT4和Nanog的表达情况。qRT-PCR检测结果显示，HCC827/GR细胞中SOX2和Nanog的mRNA表达水平显著高于HCC827细胞(*P* < 0.01)，OCT4 mRNA表达则没有明显变化(图 5A)。在HCC827/GR细胞中，干扰FOXC1能够明显下调HCC827/GR细胞SOX2的mRNA和蛋白表达水平(图 5B，图 5C)。然后，对干扰FOXC1的HCC827/GR细胞转染过表达SOX2的质粒，Western blot结果显示SOX2的表达水平升高(图 5D)。MTS实验检测发现，干扰FOXC1的HCC827/GR细胞在过表达SOX2后的增殖能力相比未过表达SOX2的细胞有所提高(*P* < 0.05，图 5E)。同时，干扰FOXC1的HCC827/GR细胞过表达SOX2后相比表达SOX2前的IC_50_值也有所升高(*P* < 0.05，图 5F)。上述结果表明，干扰FOXC1能够抑制HCC827/GR细胞SOX2的表达。

**图 5:**
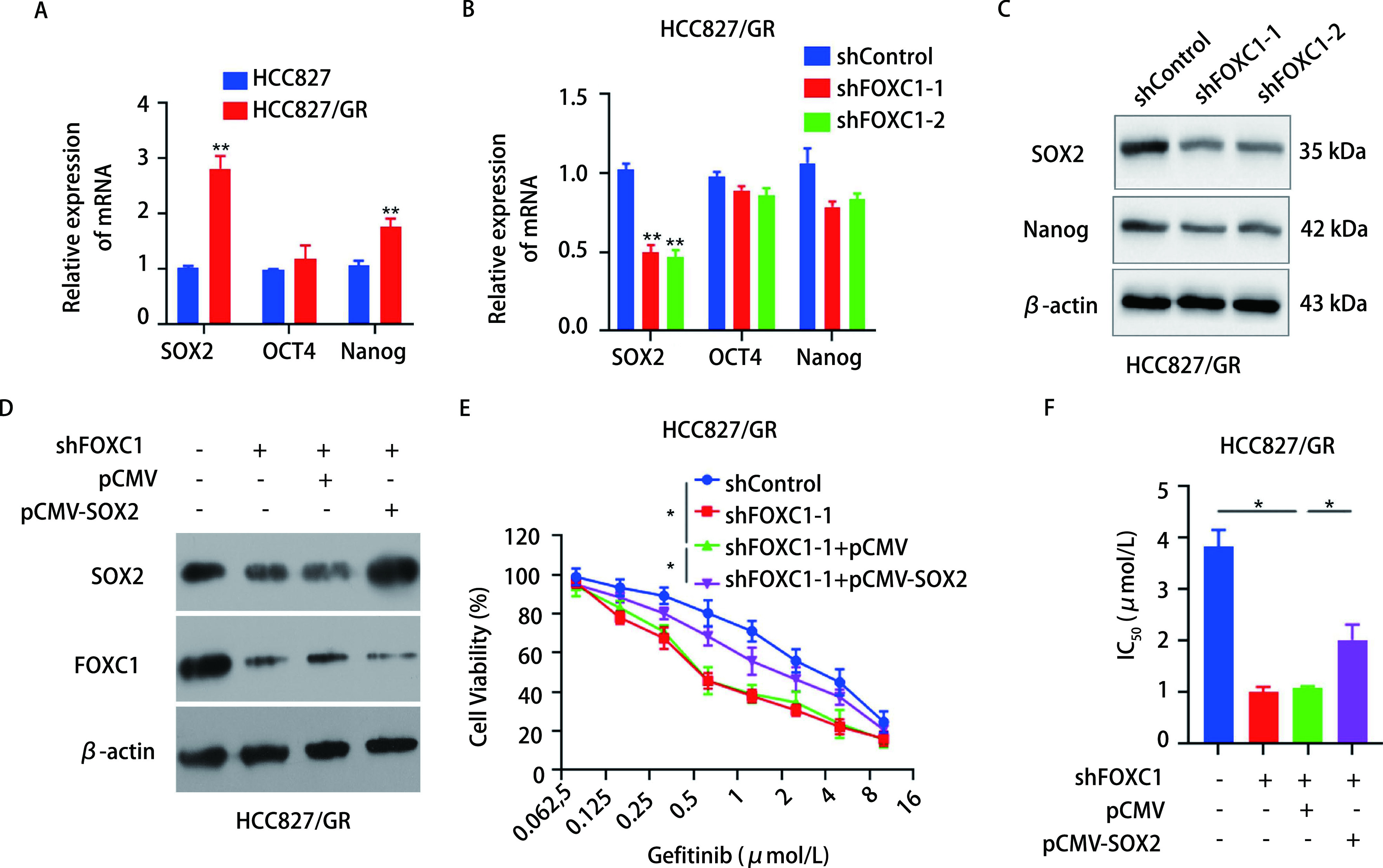
干扰FOXC1抑制HCC827/GR细胞SOX2的表达。A：qRT-PCR检测HCC827/GR细胞SOX2、OCT4和Nanog的mRNA表达水平(^**^P < 0.01);B：qRT-PCR检测干扰FOXC1后HCC827/GR细胞SOX2、OCT4和Nanog的mRNA表达水平(^**^P < 0.01);C：Western blot检测干扰FOXC1后HCC827/GR细胞SOX2和Nanog的蛋白表达水平; D-F：使用干扰FOXC1的HCC827/GR细胞，构建稳定过表达SOX2的细胞株; D：Western blot检测SOX2和FOXC1的蛋白表达水平; E：MTS检测细胞增殖能力(^*^P < 0.05);F：计算IC_50_值(^*^P < 0.05)。 Knockdown of FOXC1 inhibits the expression of SOX2 in HCC827/GR cells. A: The mRNA levels of SOX2, OCT4 and Nanog in HCC827/GR cells were detected by qRT-PCR (^**^P < 0.01); B: The mRNA levels of SOX2, OCT4 and Nanog in HCC827/GR cells with FOXC1 knockdown were detected by qRT-PCR (^**^P < 0.01); C: The protein levels of SOX2 and Nanog in HCC827/GR cells with FOXC1 knockdown were detected by Western blot; D-F: HCC827/GR cells with FOXC1 knockdown were stably transfected with pCMV-SOX2 and with an empty vector as a control; D: The protein levels of SOX2 and FOXC1 were detected by Western blot; E: MTS assays were used to detect cell viability (^*^P < 0.05); F: The IC_50_ of cells were calculated (^*^P < 0.05).

### 2.6 FOXC1、SOX2和CD133在吉非替尼耐药组织中表达的相关性

为进一步从体内验证FOXC1、SOX2和CD133表达的关系，采用免疫组化检测15例吉非替尼耐药组织和15例吉非替尼敏感组织中FOXC1、CD133和SOX2的表达情况。SOX2和CD133在NSCLC吉非替尼耐药组织中的表达均显著高于敏感组织(*P* < 0.01，图 6A)。FOXC1高表达的NSCLC组织中，同时伴随着SOX2与CD133的高表达; FOXC1低表达的组织中，SOX2与CD133的表达量则偏低(图 6B，图 6C)。同时，选取了癌症基因图谱(The Cancer Genome Atlas, TCGA)数据库中肺腺癌患者组织FOXC1、CD133和SOX2表达的数据，探讨三个基因的相关性。如图 6D所示，FOXC1、SOX2和CD133的表达均两两呈正相关(*P* < 0.05)。以上结果表明，FOXC1、SOX2和CD133在吉非替尼耐药组织中表达两两呈正相关。

**图 6:**
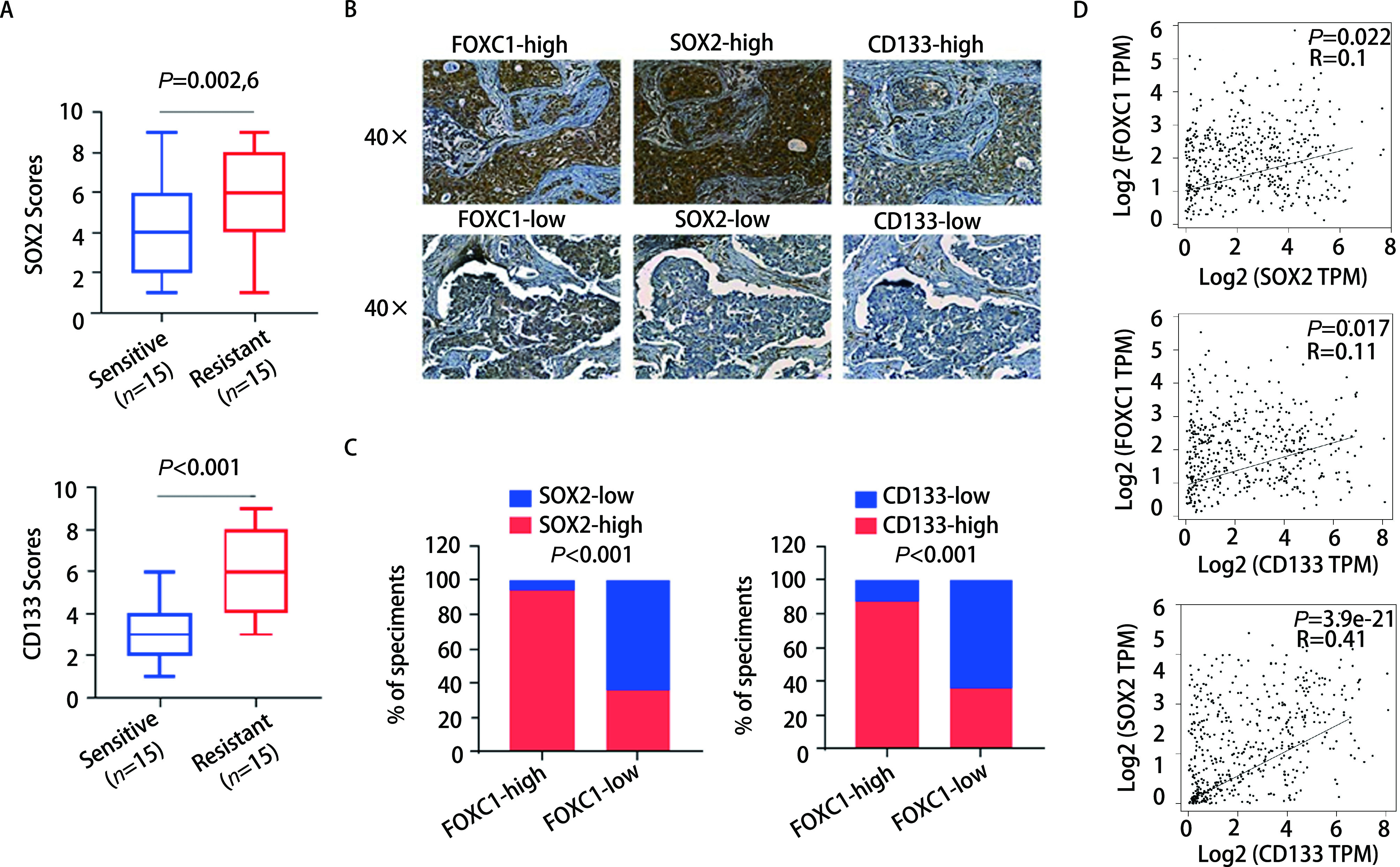
FOXC1、CD133和SOX2在吉非替尼耐药组织中的表达相关性分析。A：箱线图分析SOX2和CD133在NSCLC耐药组织表达的得分情况; B：同一NSCLC组织中FOXC1、SOX2和CD133的表达情况; C：NSCLC组织中FOXC1、SOX2和CD133表达差异的分布情况; D：基于TCGA数据库的肺腺癌数据集，分析FOXC1、SOX2和CD133表达的相关性。 Correlations of the expressions of FOXC1, CD133 and SOX2 were assessed in NSCLC tissues with gefitinib resistance. A: SOX2 and CD133 expression scores in NSCLC tissues with gefitinib resistance; B: Representative examples of the FOXC1, SOX2 and CD133 staining in the same NSCLC tissue set; C: Differences of expressions of FOXC1, SOX2 and CD133 in NSCLC tissues; D: Correlations of the expressions of FOXC1, CD133 and SOX2 with each other in lung adenocarcinoma samples from TCGA database.

## 3 讨论

EGFR-TKI靶向治疗以其选择性作用强，疗效显著，毒副作用小等优势在临床NSCLC治疗中越来越受到重视。吉非替尼是首个口服的EGFR-TKI，研究^[12]^发现对于*EGFR*突变阳性的NSCLC患者，吉非替尼一线治疗优于标准方案化疗。然而吉非替尼的有效维持时间仅为8个月-10个月，且多数患者容易出现复发，提示此类药物存在较严重的获得性耐药。目前有关EGFR-TKIs获得性耐药的主要机制包括T790M突变^[13]^、*c-MET*癌基因扩增^[14]^、上皮间质表型转化^[15]^等，然而TKI耐药是多因素参与的复杂过程，仍有约30%患者的耐药原因尚不明确。

FOXC1是叉头框蛋白家族重要成员，参与调节多种生物过程，包括发育、分化、增殖、迁移和肿瘤发生等。近年来，*FOXC1*被证实在多种癌症如乳腺癌、肝癌、前列腺癌中扮演癌基因的角色。本研究首次发现FOXC1在NSCLC吉非替尼耐药细胞和组织中显著高表达。MTS结果可见，在吉非替尼的作用下，干扰FOXC1能够明显抑制耐药细胞HCC827/GR的增殖能力(*P* < 0.05)，且IC_50_值也发生显著降低，表明干扰FOXC1能够增加HCC827/GR细胞对吉非替尼敏感性。以上结果提示，FOXC1的过表达可能是导致NSCLC患者对吉非替尼发生耐药的原因之一。

有研究^[11]^表明，肿瘤细胞中存在一小部分分化程度低、自我更新能力和成瘤能力强的CSCs，参与肿瘤生长、转移和耐药。EGFR-TKI耐药与CSCs密切相关^[16]^，在NSCLC患者的靶向治疗过程中，CSCs富集可能是获得EGFR-TKI耐药性的重要原因^[17]^。研究^[18]^发现NSCLC吉非替尼耐药细胞具有CSCs特性，高表达CSCs相关基因，自我更新能力强。CD133是人NSCLC中CSCs的特定表面标记^[19]^。根据TCGA数据库的数据进行分析，人肺腺癌组织中FOXC1与CD133表达呈正相关。我们的实验结果发现干扰FOXC1能够抑制HCC827/GR细胞CD133的表达，说明FOXC1参与了对CSCs特性的调控。此外，微球体形成实验表明干扰FOXC1能够使HCC827/GR细胞的自我更新能力受到抑制。OCT4、NANOG和SOX2是干性相关的调控基因，可帮助NSCLC维持CSCs样特性^[20, 21]^，而干扰FOXC1可显著降低SOX2的表达。再者，HCC827/GR细胞干扰FOXC1之后过表达SOX2，SOX2表达水平升高且细胞增殖能力增强，进一步说明FOXC1通过调节SOX2表达来促进NSCLC吉非替尼耐药细胞的CSCs样特性，维持细胞耐药性。Cao等^[22]^研究发现，FOXC1通过促进β-catenin表达进而增强NSCLC的CSCs样特性。另有研究^[23]^指出，SOX2是受β-catenin调控的靶基因。因此，我们推断β-catenin介导FOXC1对SOX2的表达调控。以上结果表明，FOXC1可能通过调控CSCs样特性促进NSCLC吉非替尼耐药。

总之，我们研究发现FOXC1在NSCLC吉非替尼耐药中起到重要作用，是逆转NSCLC吉非替尼耐药的有效靶点。而FOXC1对CSCs的调控作用可能是其促进NSCLC吉非替尼耐药的重要机制。
